# Identifying Critical Points of Trajectories of Depressive Symptoms from Childhood to Young Adulthood

**DOI:** 10.1007/s10964-018-0976-5

**Published:** 2019-01-22

**Authors:** Alex S. F. Kwong, David Manley, Nicholas J. Timpson, Rebecca M. Pearson, Jon Heron, Hannah Sallis, Evie Stergiakouli, Oliver S. P. Davis, George Leckie

**Affiliations:** 1 School of Geographical Sciences, University of Bristol, University Road, Bristol, BS8 1SS UK; 2 Centre for Multilevel Modelling, University of Bristol, Bristol, UK; 3 MRC Integrative Epidemiology Unit at the University of Bristol, Bristol, UK; 4 Population Health Sciences, Bristol Medical School, University of Bristol, Bristol, UK; 5 Centre for Academic Mental Health at the University of Bristol, Bristol, UK; 6 UK Centre for Tobacco and Alcohol Studies, School of Experimental Psychology, University of Bristol, Bristol, UK; 7 Oral and Dental Sciences, University of Bristol, Bristol, UK; 8 School of Education, University of Bristol, Bristol, UK

**Keywords:** Depressive symptoms, Trajectories, Multilevel growth-curve modelling, ALSPAC, Critical points

## Abstract

Depression is a common mental illness and research has focused on late childhood and adolescence in an attempt to prevent or reduce later psychopathology and/or social impairments. It is important to establish and study population-averaged trajectories of depressive symptoms across adolescence as this could characterise specific changes in populations and help identify critical points to intervene with treatment. Multilevel growth-curve models were used to explore adolescent trajectories of depressive symptoms in 9301 individuals (57% female) from the Avon Longitudinal Study of Parents and Children, a UK based pregnancy cohort. Trajectories of depressive symptoms were constructed for males and females using the short mood and feelings questionnaire over 8 occasions, between 10 and 22 years old. Critical points of development such as age of peak velocity for depressive symptoms (the age at which depressive symptoms increase most rapidly) and the age of maximum depressive symptoms were also derived. The results suggested that from similar initial levels of depressive symptoms at age 11, females on average experienced steeper increases in depressive symptoms than males over their teenage and adolescent years until around the age of 20 when levels of depressive symptoms plateaued and started to decrease for both sexes. Females on average also had an earlier age of peak velocity of depressive symptoms that occurred at 13.5 years, compared to males who on average had an age of peak velocity at 16 years old. Evidence was less clear for a difference between the ages of maximum depressive symptoms which were on average 19.6 years for females and 20.4 for males. Identifying critical periods for different population subgroups may provide useful knowledge for treating and preventing depression and could be tailored to be time specific for certain groups. Possible explanations and recommendations are discussed.

## Introduction

Depression is a common mental illness believed to affect more than 300 million people worldwide (WHO 2017). Depression is comorbid with other psychiatric conditions including anxiety, bipolar disorder and schizophrenia (Malhi and Mann 2018; Thapar et al. 2012), and is often associated with increased substance misuse, impaired educational attainment and increased risk of suicide (Fergusson et al. 2007; Marmorstein 2009).

One avenue for research has focused on childhood and adolescent depressive symptoms as a potentially modifiable risk factor for more severe depression during adulthood (Hill et al. 2014; Musliner et al. 2016) and previous research has shown that elevated levels of depressive symptoms within a population (and even those that never rise above the threshold for depression) are associated with a greater risk for depression in later life (Ellis et al. 2017; Yaroslavsky et al. 2013). Evidence concludes that the period between late childhood and adolescence may be important for subsequent mental health and social functioning, thus identifying and treating elevated depressive symptoms during this time could limit or prevent depression in later life through support and services (Fergusson et al. 2005; Thapar et al. 2012).

Research has yet to fully understand the nature of trajectories of depressive symptoms across adolescence and into young adulthood and identifying the key characteristics and time course of depressive symptoms across this period will ultimately aid in improving treatments and interventions. The current study attempts to build upon previous research by estimating trajectories of depressive symptoms between late childhood and young adulthood, and identifying critical points along those trajectories that could shed light on the nature of how these trajectories of depressive symptoms develop and change over time.

Previous studies have examined trajectories of depressive symptoms between childhood and young adulthood in an attempt to explore the nature and risk factors underlying greater depressive symptoms, for reviews see Musliner et al. (2016), Shore et al. (2018) and Schubert et al. (2017). Indeed, research suggests that trajectories of depressive symptoms tend to increase from childhood through adolescence, before decreasing in young adulthood (Ge et al. 2006; Natsuaki et al. 2009). Evidence also suggests that these trajectories may peak in mid-to-late adolescence, towards the ages of 15–17 years old (Costello et al. 2008; Ferro et al. 2015b).

Other research has suggested that depressive symptoms may increase post adolescence. For instance, Ge et al. (2001) demonstrated that trajectories of depressive symptoms were highest around the ages of 17–18 years old, but were unable to explore depressive symptoms further than this age due to a cease in data collection. Other research has suggested that trajectories of depressive symptoms are highest in young adulthood (towards age of 20) but then decline until older age (Sutin et al. 2013). In this instance, Sutin and colleagues did not have data preceding young adulthood so they were unable to explore if trajectories were higher around adolescence. It may be that trajectories of depressive symptoms could peak outside adolescence, yet do not have preceding and succeeding data around this period to substantiate these claims (Brendgen et al. 2005; St Clair et al. 2012). Likewise, many studies also use small sample sizes, which can make it difficult to infer about population level changes of depressive symptoms.

Observing the longitudinal nature of trajectories of depressive symptoms enables researchers to identify critical points at which to intervene to limit or prevent more severe depression. The notion of identifying critical points is not new (Bartley et al. 1997), but this application to trajectories of depressive symptoms has yet to be fully explored. Using depressive symptoms data from childhood to young adulthood (12 to 25 years), Ferro et al. (2015b) identified the age of maximum depressive symptoms to occur between the ages of 15 to 17 years old, whilst Rawana and Morgan (2014) found that depressive symptoms were highest at approximately 17 years old. Similar results have also been observed by Natsuaki et al. (2009) who plotted trajectories that indicated the age of maximum depressive symptoms was 16 years old. Other research has also found evidence of sex differences in regards to an age of maximum depressive symptoms with females reaching this maximum earlier than males (Adkins et al. 2009; Edwards et al. 2014).

While identifying the age of maximum depressive symptoms is important for characterising the nature of depressive symptoms, calculation of the age of peak velocity may also be important (i.e., the age at which depressive symptoms are increasing most rapidly). Research has suggested that identifying depressive symptoms early or before depression already manifested may be a key step towards preventing greater depressive symptoms or severe depression from occurring (Cuijpers and Smit 2004; Kessler et al. 2001). By identifying the age of peak velocity of depressive symptoms, it may be possible to highlight a critical point where interventions and treatments could be implemented to reduce or limit greater depressive symptoms from escalating. However, no studies have calculated the age of peak velocity of depressive symptoms and studies that have previously identified the age of maximum depressive symptoms have done so using heuristics, graphs or figures rather than empirically calculating these ages.

Trajectories of depressive symptoms are not homogeneous within the population (Musliner et al. 2016), and a number of risk factors can influence trajectories of depressive symptoms (Weeks et al. 2014; Yaroslavsky et al. 2013). Consistent evidence has shown that females tend to have higher trajectories of depressive symptoms compared to males (Dekker et al. 2007; Yaroslavsky et al. 2013). Although in several studies where heterogeneous trajectories are identified, there is some evidence that females are associated with more intermediary trajectories such as “increasing”, “late onset” or “early high” (Costello et al. 2008; Olino et al. 2010). These studies suggest there may be mechanisms that underpin membership into varying trajectories, but the extent to which this can be explained has yet to be fully understood. Females also appear to have a higher peak along these trajectories of depressive symptoms (Adkins et al. 2009; Ge et al. 2001; Natsuaki et al. 2009), and may reach this peak earlier than males (Edwards et al. 2014), yet it is also not clear why this is the case.

## Current Study

Previous research has shown that trajectories of depressive symptoms tend to increase throughout adolescence and into young adulthood (Costello et al. 2008; Ferro et al. 2015b), and that females are more likely to have higher trajectories of depressive symptoms (Ge et al. 2006; Natsuaki et al. 2009). However, the longitudinal nature of depressive symptoms has yet to be fully understood as previous research has used small sample sizes, not had depressive symptoms data that spans a wide enough temporal window, and/or has been unable to measure depressive symptoms over important periods and transitions of development (e.g., from late childhood to adolescence and/or from adolescence into young adulthood). Likewise, no studies have identified critical periods of trajectories of depressive symptoms that could potentially highlight modifiable stages at which to best intervene. Identifying critical points such as the age of peak velocity of depressive symptoms (i.e., the age at which depressive symptoms are increasing most rapidly) could help researchers and clinicians target a stage of development where depressive symptoms might be increasing at the fastest rate, which may in turn prevent or reduce higher symptoms or the onset of depression. The current study aims to address the previous limitations firstly by using multilevel growth-curve modelling to create trajectories of depressive symptoms in a large UK based population cohort. Multilevel growth-curve modelling has previously been a useful method for examining trajectories of depressive symptoms (Ferro et al. 2015a; Rawana and Morgan 2014). Trajectories are created for both male and female populations between 10 and 22 years using 8 waves of depressive symptoms follow up. It was hypothesised that females would have higher trajectories of depressive symptoms throughout adolescence and young adulthood compared to males. Secondly, the ages of peak velocity and maximum depressive symptoms, as well as the depressive symptoms scores at both these ages for males and females are then calculated and compared. It was also hypothesised that the ages of peak velocity and maximum depressive symptoms would be earlier for females given their tendency to have elevated trajectories at the beginning of adolescence and that they commence on these trajectories earlier than males.

## Methods

### Participants

Participants were from the Avon Longitudinal Study of Parents and Children (ALSPAC), an ongoing population-based study in the South-West of England designed to examine the effects of a number of factors on health and development. ALSPAC recruited pregnant mothers with an estimated delivery date between April 1991 and December 1992. The initial cohort consisted of 14,541 pregnancies, with 13,988 children still alive one year later (52% males and 48% females). ALSPAC children were slightly more educated at age 16 compared to the national average, slightly more likely to come from homeowner backgrounds and slightly more likely to be of Caucasian descent (Boyd et al. 2013). Ethical oversight for the study was obtained from the ALSPAC Law and Ethics Committee and Local Research Ethics Committees. Participant data has been collected on the mothers, fathers and children from early pregnancy, and measured via questionnaires and regular clinic visits. Part of this data was collected using REDCap (https://projectredcap.org/resources/citations/). Detailed information about ALSPAC is available on the study website (www.bris.ac.uk/alspac), which also contains a fully searchable data dictionary (www.bris.ac.uk/alspac/researchers/data-access/data-dictionary). See Table 1 for participant characteristics.

**Table 1:** Descriptive statistics and reliability of the short mood and feelings questionnaire (SMFQ)

Occasion	Mean age	Sample size	Mean SMFQ	SMFQ SD	Above SMFQ threshold (<11)	α	Source of SMFQ
1	10.65	7335	4.04	3.51	5.4%	0.797	Clinic
2	12.81	6692	3.97	3.86	7.08%	0.842	Clinic
3	13.84	5996	4.92	4.49	11.64%	0.865	Clinic
4	16.68	4977	5.91	5.64	18.08%	0.908	Questionnaire
5	17.84	4486	6.59	5.25	21.67%	0.897	Clinic
6	18.65	3323	6.83	5.93	21.88%	0.906	Questionnaire
7	21.95	3293	5.7	5.58	18.07%	0.915	Questionnaire
8	22.88	3840	6.21	5.55	18.83%	0.906	Questionnaire

α: Coefficient alpha estimate of reliability for the SMFQ at each occasion. The SMFQ ranges between 0–26 and scores exceeding 11 have been proposed as good markers for depression (see Turner et al. 2014)

### Measures

#### Depressive symptoms

The main outcome measure was depressive symptoms, assessed using the short mood and feelings questionnaire (SMFQ) (Angold et al. 1995). The SMFQ is a 13-item questionnaire that measures the occurrence of depressive symptoms over the preceding two weeks with higher scores indicating more severe depressive symptoms (range 0–26). The SMFQ correlates highly with clinical measures of depression (Turner et al. 2014), and has been used in previous studies examining trajectories of depressive symptoms (Kingsbury et al. 2016; Mahedy et al. 2017). Self-report SMFQ data was used from eight occasions beginning in late childhood and extending to young adulthood. There was good reliability for the depressive symptoms scores used in this sample (α = 0.79–0.91). Questionnaires were either completed via postal/internet questionnaires or by computer at a clinic visit. See Table 1 for SMFQ descriptive and reliability statistics.

#### Biological sex

Biological sex was identified from birth notifications around the time of delivery and coded as a dummy variable for being female (male = 0; female = 1). Participants were removed from the analysis if sex was unknown. See Table 2 for sex specific SMFQ descriptive statistics.

**Table 2:** Descriptive statistics for the short mood and feelings questionnaire (SMFQ) by sex

Occasion	Male sample size	Female sample size	Mean male SMFQ	Male SMFQ SD	Mean female SMFQ	Female SMFQ SD
1	3605	3730	4.17	3.45	3.92	3.91
2	3271	3421	3.57	3.47	4.35	4.16
3	2922	3074	4.09	3.8	5.71	4.93
4	2010	2967	4.31	4.58	7.0	6.02
5	1898	2588	5.63	4.77	7.29	5.47
6	1172	2151	5.34	5.02	7.64	6.21
7	1161	2132	4.87	4.82	6.15	5.90
8	1323	2517	5.35	4.95	6.67	5.79

### Statistical Methods

Trajectories of depressive symptoms were estimated using multilevel growth-curve modelling (Bryk and Raudenbush 1987; Steele 2008). Descriptive statistics indicated the change in depressive symptoms followed a non-linear pattern (Table 1). Thus the model needed to appropriately capture the non-linearity of these trajectories. A multilevel cubic growth-curve polynomial model was chosen for two reasons. First, entering age as a cubic polynomial fitted the data better and produced more plausible results than entering age as a quadratic or quartic polynomial (see Supplementary Table 3). Second, compared to more complex cubic splines and fractional polynomials which have also been used to model non-linear growth in other contexts (Grajeda et al. 2016; Howe et al. 2016), entering age as a cubic polynomial led to a more parsimonious model. Multilevel cubic growth-curve polynomials have been used in previous studies examining trajectories of depressive symptoms (Ferro et al. 2015a; Rawana and Morgan 2014). Model fit was assessed using likelihood ratio tests and information criteria, consistent with other studies using multilevel growth-curve models (Rawana and Morgan 2014; Singer and Willett 2003). The preferred model specifies separate population-averaged trajectories for males and females by interacting the fixed-effects age polynomial terms with the female dummy variable. The age polynomial terms were allowed to vary randomly across individuals to capture each individual’s unique trajectory. The model can be written as

1 $$\begin{array}{l}y_{ij} = \beta _0 + \beta _1t_{ij} + \beta _2t_{ij}^2 + \beta _3t_{ij}^3 + \beta _4x_{1j} + \beta _5x_{1j}t_{ij} + \beta _6x_{1j}t_{ij}^2\\ +\, \beta _7x_{1j}t_{ij}^3 + u_{0j} + u_{1j}t_{ij} + u_{2j}t_{ij}^2 + u_{3j}t_{ij}^3 + e_{ij}\end{array}$$

where *y*_*ij*_ is the depressive symptom score and *t*_*ij*_ is the age^1^ for individual *j* at occasion *i*, *x*_1*j*_ is a dummy variable for being female, and *u*_0*j*_, *u*_1*j*_, *u*_2*j*_, and *u*_3*j*_ are the random intercept, linear, quadratic and cubic effects of age, respectively. The occasion-specific residual *e*_*ij*_ allows the depressive symptom scores to deviate from the individual-specific cubic trajectories.

The random effects are assumed multivariate normal distributed with zero mean vector and constant covariance matrix

2 $$\left( {\begin{array}{*{20}{c}} {u_{0j}} \\ {u_{1j}} \\ {u_{2j}} \\ {u_{3j}} \end{array}} \right)\sim N\left\{ {\left( {\begin{array}{*{20}{c}} 0 \\ 0 \\ 0 \\ 0 \end{array}} \right),\left( {\begin{array}{*{20}{c}} {\sigma _{u0}^2} & {} & {} & {} \\ {\sigma _{u01}} & {\sigma _{u1}^2} & {} & {} \\ {\sigma _{u02}} & {\sigma _{u12}} & {\sigma _{u2}^2} & {} \\ {\sigma _{u02}} & {\sigma _{u13}} & {\sigma _{u23}} & {\sigma _{u3}^2} \end{array}} \right)} \right\}$$

The elements of the covariance matrix summarise the degree to which individual-specific trajectories vary around the sex-specific population-averaged trajectories. The residuals are assumed normally distributed with zero mean and sex specific variance

3 $$e_{ij}\sim N\left( {0,\sigma _{ej}^2} \right)\,where\,\sigma _{ej}^2 = \alpha _0\left( {1 - x_{1j}} \right) + \alpha _1x_{1j}$$

All analyses were conducted using Stata 14 (StataCorp, College Station, TX, USA) using the user-written runmlwin command (Leckie and Charlton 2012), which calls the standalone multilevel modelling package MLwiN v2.35 (www.cmm.bristol.ac.uk/MLwiN/index.shtml). Stata code is provided in the supplementary materials for reproducibility and extension of this work.

#### Trajectory features

The population male and female trajectories are therefore given by

$$\begin{array}{*{20}{c}}{Male:}&{E({y_{ij}}|{t_{ij}},{x_{1j}} = 0)}& = &{{\beta _0} + {\beta _1}{t_{ij}} + {\beta _2}t_{ij}^2 + {\beta _3}t_{ij}^3} \\{Female:}&{E({y_{ij}}|{t_{ij}},{x_{1j}} = 1)}& = &{({\beta _0} + {\beta _4}) + ({\beta _1} + {\beta _5}){t_{ij}} + ({\beta _2} + {\beta _6})t_{ij}^2 + ({\beta _3} + {\beta _7})t_{ij}^3}\end{array}$$

Several features were calculated from these sex-specific mean trajectories including: the ages of peak velocity of depressive symptoms (i.e., ages at which male and female depressive symptoms are increasing most rapidly), and the ages of maximum depressive symptoms (i.e., the ages at which male and female depressive symptoms were the highest). The depressive symptoms scores were also calculated at each of these critical ages. The delta method in Stata was used to compare differences in the intercept, linear, quadratic and cubic terms, as well as at the critical points between the male and female trajectories. See Supplemental Materials for further details.

## Results

There were 9301 individuals with data on sex and at least one measurement of the SMFQ, resulting in 39,942 measurements (17,362 male [43.47%]/22,580 female [56.53%]).

### Trajectories of Depressive Symptoms

**Fig. 1:**
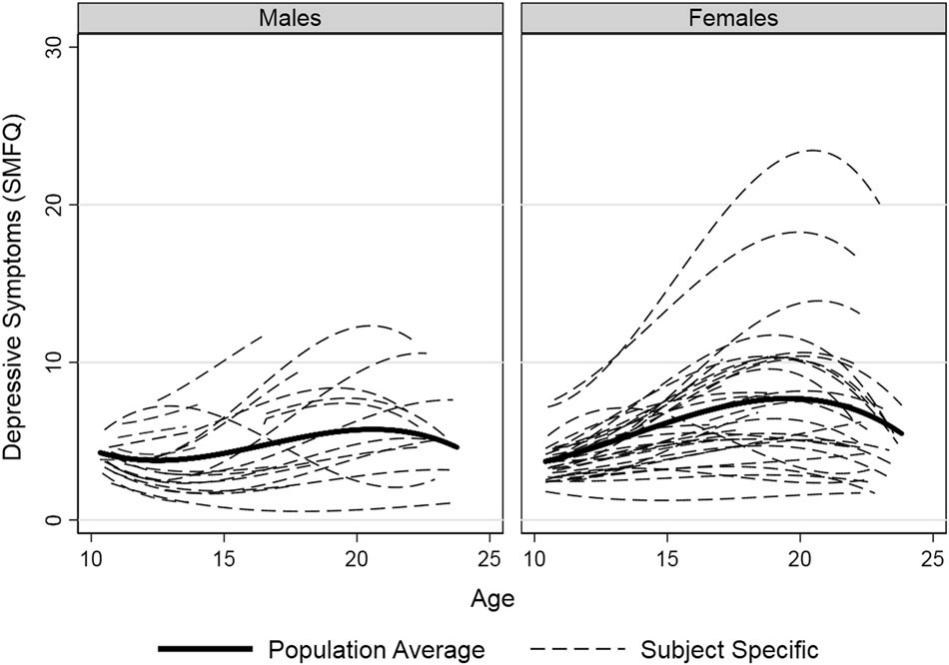
Individual and averaged population trajectories for a random set of 100 participants. SMFQ short mood and feelings questionnaire

Females and males followed different trajectories (Fig. 1). The intraclass correlation evaluated at age 16 was 0.56 (SE = 0.01) indicating that over half of the variation in individuals’ depressive symptoms at this age is captured by their individual-specific cubic trajectories. The correlation between the intercept (i.e., first measurement of depressive symptoms) and the slope (i.e., change in depressive symptoms with every year increase) was 0.52 (SE = 0.03). Table 3 presents the regression coefficients from the cubic polynomial model. The marginal sex-specific trajectories did not differ substantively with the addition of covariates (see Supplementary materials), so only the unadjusted trajectories and main features of the trajectories are described.

**Table 3:** Regression coefficients for the cubic polynomial model

	Cubic Polynomial Model (*n* = 9301)		
Parameter	Estimate	Std. Error	*p*-value
*β*_0_—Intercept	4.57 [4.43, 4.7]	0.07	<0.001
*β*_1_—Age (slope)	0.37 [0.31, 0.4]	0.027	<0.001
*β*_2_—Age^2^ (acceleration)	0.01 [0.008, 0.02]	0.002	<0.001
*β*_3_—Age^3^ (cubic change)	−0.007 [−0.009, −0.006]	0.001	<0.001
*β*_4_—Female	2.13 [1.95, 2.32]	0.1	<0.001
*β*_5_—Female × Age	0.14 [0.06, 0.19]	0.03	<0.001
*β*_6_—Female × Age^2^	−0.05 [−0.06, −0.05]	0.003	<0.001
*β*_7_—Female × Age^3^	0.002 [0.0001, 0.003]	0.001	0.04
ICC	0.56		
Deviance	227,890.58		

95% Confidence intervals given in [parenthesis]

**Fig. 2:**
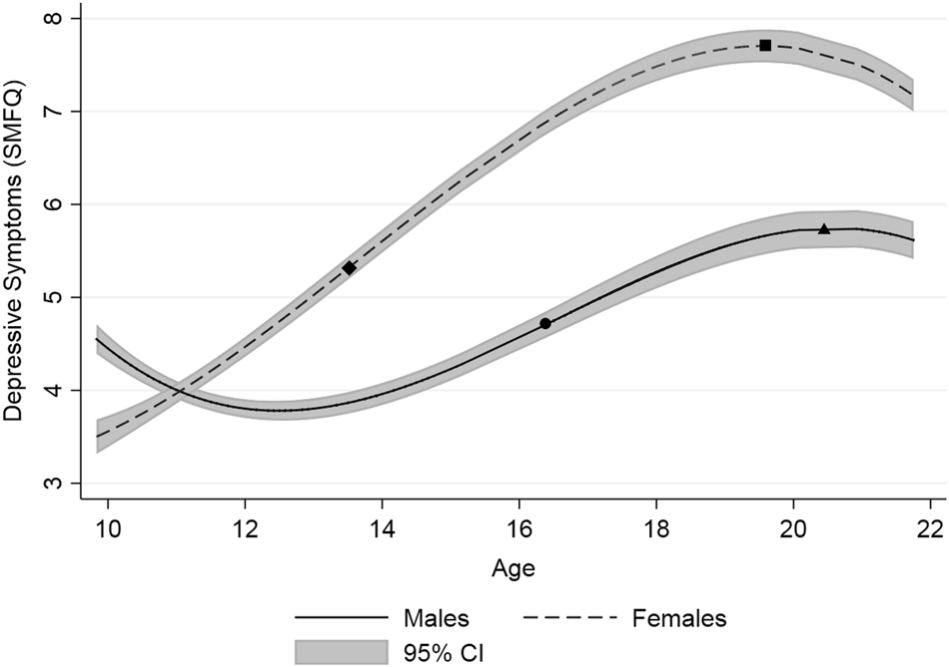
Averaged population trajectories for males and females. SMFQ short mood and feelings questionnaire. Features of the trajectories are overlaid with the following terms: ● Male age of peak velocity of depressive symptoms. ▲ Male age of maximum depressive symptoms. ♦ Female age of peak velocity of depressive symptoms. ■ Female age of maximum depressive symptoms

The differences in trajectories of depressive symptoms between males and females are shown in Table 4 and Fig. 2. Briefly, females had higher trajectories compared to males, except between 10 and 11 years old where males were predicted to have higher depressive symptoms. Females were associated with higher depressive symptoms at 16 years of age (*β* = 6.7, SE = 0.07 [95% CI: 6.57, 6.83]) compared to males (*β* = 4.57, SE = 0.07 [95% CI: 4.43, 4.7]; *p*^diff^ < 0.001). Females had a higher linear slope (*β* = .049, SE = 0.02 [95% CI: 0.45, 0.53]) compared to males (*β* = 0.36, SE = 0.02 [95% CI: 0.31, 0.4]; *p*^diff^ < 0.001), which suggested that depressive symptoms increased more rapidly for females until the age of 20. However, females had a greater negative quadratic slope (*β* = −0.04, SE = 0.002 [95% CI: −0.05, −0.03]), implying from around age 20 their depressive symptoms started to decrease, in contrast to males (*β* = 0.01, SE = 0.002 [95% CI: 0.008, 0.2]; *p*^diff^ < 0.001). Finally, a negative cubic slope was observed for both females (*β* = −0.01, SE = 0.001 [95% CI: −0.001, −0.004]) and males (*β* = −0.007, SE = 0.001 [95% CI: −0.01, −0.006]; *p*^diff^ = 0.04), indicating that depressive symptoms were decreasing from age 20 onwards, however the current analysis suggested that both sexes decreased at similar rates into young adulthood (Tables 3 and 4).

**Table 4:** Comparing parameter estimates and trajectories from the cubic polynomial model

	Males	Females	Difference	*p-*value
Intercept term for SMFQ	4.57 (0.07)	6.7 (0.07)	2.13 (0.1)	<0.001
	[4.43, 4.7]	[6.57, 6.83]	[1.95, 2.32]	
Linear term for SMFQ	0.36 (0.02)	0.49 (0.02)	0.14 (0.03)	<0.001
	[0.31, 0.4]	[0.45, 0.53]	[0.08, 0.19]	
Quadratic term for SMFQ	0.01 (0.002)	−0.04 (0.002)	0.05 (0.003)	<0.001
	[0.008, 0.2]	[−0.05, −0.03]	[0.05, 0.06]	
Cubic term for SMFQ	−0.007 (0.001)	−0.01 (.001)	0.002 (0.001)	0.04
	[−0.01, −0.006]	[−0.001, −0.004]	[0.0001, 0.003]	

The intercept was centered to age 16 for interpretability. The differences between each term were calculated as follows: the intercept term for males (*β*_0_) *minus* the intercept term for females (*β*_0_ + *β*_4_), the linear term for males (*β*_1_) *minus* the linear term for females (*β*_1_ + *β*_5_), the quadratic term for males (*β*_2_) *minus* the quadratic term for females (*β*_2_+*β*_6_), the cubic term for males (*β*_3_) *minus* the cubic term for females (*β*_3_+*β*_7_). Standard errors are given in (parenthesis), 95% confidence intervals are given in [parenthesis]

### Age of Peak Velocity and Age at Maximum Depressive Symptoms

The age of peak velocity in the model was earlier for females (13.51 years old, SE = 0.32 [95% CI: 12.88, 14.14]) compared to males (16.36 years old, SE = 0.1 [95% CI: 16.18, 16.55]; *p*^diff^ < 0.001). However there was little evidence of a difference between the age at the maximum point of depressive symptoms for females (19.61 years old, SE = 0.5 [95% CI: 18.63, 20.6]) compared to males (20.42 years old, SE = 0.14 [95% CI: 20.14, 20.69]; *p*^diff^ = 0.13). See Table 5 for full statistics.

**Table 5:** Calculated features from the trajectories of the cubic polynomial model

	Males	Females	Difference	*p*-value
Age of peak velocity in SMFQ	16.36 (0.1)	13.51 (0.32)	2.86 (0.34)	<0.001
	[16.18, 16.55]	[12.88, 14.14]	[2.2, 3.51]	
Age of maximum SMFQ	20.42 (0.14)	19.61 (0.5)	0.80 (0.55)	0.14
	[20.14, 20.69]	[18.63, 20.6]	[−0.27, 1.88]	
SMFQ at peak velocity	4.76 (0.07)	5.42 (0.06)	0.66 (0.1)	<0.001
	[4.62, 4.91]	[5.3, 5.55]	[0.47, 0.85]	
SMFQ at maximum point	5.75 (0.1)	7.7 (0.09)	1.95 (0.14)	<0.001
	[5.55, 5.95]	[7.52, 7.88]	[1.69, 2.22]	

Standard errors are given in (parenthesis) 95% confidence intervals are given in [parenthesis]

### Depressive Symptoms Score at Age of Peak Velocity and at Age of Maximum Point of Depressive Symptoms

The predicted depressive symptoms scores at the estimated age of peak velocity were higher for females (5.42 points, SE = 0.06 [95% CI: 5.3, 5.55]) than they were for males (4.76 points, SE = 0.07 [95% CI: 4.62, 4.91]; *p*^diff^ < 0.001). The depressive symptoms scores at the maximum point of depressive symptoms were also higher for females (7.7 points, SE = 0.09 [95% CI: 7.52, 7.88]) compared to males (5.75 points, SE = 0.1 [95% CI: 5.55, 5.95]; *p*^diff^ < 0.001). See Table 5 for full statistics.

### Missing Data and Sensitivity Analysis

Missing data were handled using full information maximum likelihood (FIML), which assumes that data are missing at random. The initial analysis included individuals with at least one measurement from the SMFQ. Although individuals with only one measurement do contribute to the analysis (e.g. via association between average depressive symptoms and sex), it is not meaningful to predict and examine trajectories for these individuals. To overcome this, a sensitivity analysis was run on a subset of individuals who had four or more depressive symptom scores. The sample size reduced from 9,301 to 5,409. However, removing these individuals had no substantive impact on the interpretation and conclusions from these trajectories (Supplementary Tables 5 and 8), thus only the full analysis is reported here. Results were also robust to the inclusion of covariates that are associated with missing data and attrition. Further analysis on individuals with depressive symptoms data at ages 10 and 22 revealed varying distributions of the depressive symptoms data and underlying demographics compared with individuals who only had depressive symptoms data at age 10 (i.e., had depressive symptoms data missing at 22), see Supplementary Table 2. Further information regarding analyses using these covariates can be found in the Supplementary Materials.

## Discussion

The longitudinal nature of trajectories of depressive symptoms between adolescence and young adulthood is not fully established, and research has yet to identify critical periods of trajectories of depressive symptoms that could potentially be used to target a stage of development where depressive symptoms might be increasing at the fastest rate. A greater understanding of the nature of depressive symptoms would aid researchers and clinicians in developing and improving treatments and interventions. The purpose of this study was to explore trajectories of depressive symptoms from childhood to young adulthood between males and females and to identify and compare critical points along these trajectories for both populations.

This study’s results showed that females and males have different population-averaged trajectories of depressive symptoms, with varying ages of peak velocity of depressive symptoms. Using multilevel growth-curve modelling on 8 waves of data between ages 11 to 22, results suggest that females were on average associated with higher trajectories of depressive symptoms compared to males, with the exception of between 10 and 11 years old where on average males had higher depressive symptoms. Both male and female population-averaged trajectories increased during adolescence before declining in young adulthood, yet females on average had a higher rate of deceleration (depressive symptoms slowing down) from age 20 and both sexes had trajectories that first plateaued and then started to decline in young adulthood. Evidence suggested that that on average, females had an earlier age of peak velocity of depressive symptoms (i.e., age at which depressive symptoms is increasing most rapidly), but little evidence to indicate that females had an earlier age of maximum depressive symptoms (i.e., age at which depressive symptoms is highest on the trajectory). Finally, depressive symptoms scores at the age of peak velocity and age of maximum depressive symptoms were both higher for females on average compared to males.

These findings support earlier hypothesis and previous research that trajectories of depressive symptoms increase from late childhood, through adolescence and begin to decrease in young adulthood (Adkins et al. 2009; Ferro et al. 2015b; Natsuaki et al. 2009). One possible explanation for increased depressive symptoms during adolescence is that young people face a number of social, psychological and biological changes during this stage of development (Adkins et al. 2009; Thapar et al. 2012). These changes include transitioning between schools, making new friends, taking exams and experiencing puberty. As many studies highlight that trajectories of depressive symptoms increase during this period, efforts should be made to monitor individuals who show heighted depressive symptoms as they may be individuals at a greatest risk of depression or higher levels of depressive symptoms.

This study was also able to expand on previous work by estimating points on population-averaged trajectories marking the average age of peak velocity, of maximum depressive symptoms and the depressive symptoms scores at both of these ages. These results show the age of peak velocity of depressive symptoms was almost 3 years earlier for females. The results suggest that depressive symptoms are increasing most rapidly for females at approximately 13.7 years old and for males at 16.4 years old, and that treatment and interventions could be implemented at different ages for males and females. These findings have implications for clinical services, schools and parents, who should be made aware that females are more likely to be younger when depressive symptoms are increasing most rapidly, with males following at a later stage. Likewise, as females appear to have higher trajectories for a longer of period of time, more awareness could be targeted towards services, schools and parents over this period of heightened and increasing depressive symptoms. Identifying features such as the age of peak velocity may help determine at what age depressive symptoms are getting worse most rapidly, but also highlight a key characteristic of trajectories of depressive symptoms that is potentially modifiable. Future research should primarily examine if the age of peak velocity identified here is universal to other cultures and countries and should also look to examine other predictors of these ages to see if they are potentially modifiable. Given that individuals with higher starting points (intercepts), had steeper trajectories (slopes), it is important to identify depressive symptoms early and when they are increasing as the current results suggest that those who start with higher depressive symptoms are at a greater risk of continuing to have higher trajectories of depressive symptoms.

This study’s findings suggested that the age of maximum depressive symptoms was approximately a year apart between males (20.7 years old) and females (19.7 years old), although evidence for a difference was weak. Several studies have suggested that this age should occur around mid-to-late adolescence, approximately between 15–17 years old (Ferro et al. 2015b; Natsuaki et al. 2009; Rawana and Morgan 2014). However, other evidence has indicated that this age of maximum depressive symptoms occurs later in development (Ge et al. 2001; Sutin et al. 2013), which coincides with the current results. There are several potential explanations as to why the current results observed a much later age of maximum depressive symptoms compared to some previous research. In several studies, the number of depressive symptoms measurements from follow-up are low and are therefore unable to pick up more nuanced changes in depressive symptoms over time. Depressive symptoms are dynamic and can change rapidly over a short period, thus researchers need frequent measurements to track subtle changes (Wesselhoeft et al. 2013). In the present study, the measurements were assessed on average never more than 3.5 years apart, which is more regular than many of the previous studies. It could be that frequently assessing depressive symptoms allowed detected changes and characteristics of the trajectories that were not observed in previous studies.

Another explanation is that variation in the age of maximum depressive symptoms is the result of contextual differences between the cohorts used. For example, similar ages of maximum depressive symptoms were observed using data from the National Longitudinal Study of Adolescents Health (Costello et al. 2008; Natsuaki et al. 2009), and the Canadian-based National Longitudinal Survey of Children and Youth (Ferro et al. 2015b; Rawana and Morgan 2014). However, other research using different North American cohorts have observed later ages of maximum depressive symptoms (Ge et al. 2001; Sutin et al. 2013). ALSPAC is one of the few longitudinal studies in the UK that currently has repeat depressive symptoms data across this period of development so it hard to conclude whether similar effects would be observed in other UK studies at this point. Other studies that examine depressive symptoms in longitudinal settings around the world use alternative methods to derive latent classes of trajectories of depressive symptoms (Costello et al. 2008; Yaroslavsky et al. 2013), and as such it is harder to compare critical points from these studies as there are often 4 or 5 trajectories from each study (each with their own age of maximum depressive symptoms). However, an important point to consider is that this study is the first to empirically estimate this age and present it with measures of uncertainty rather than simply describing it from figures. It is unlikely that this will explain the observed differences with other studies, but in the interest of interpretability and to help characterise the nature of trajectories of depressive symptoms, future research should estimate and report this age as this will help clarify whether there cross cultural differences for critical ages in the development of depression. Similarly, this is the first study to calculate and then compare critical points from two population trajectories (males vs. females). Interestingly, the current results supported previous research that suggested no sex differences between the ages of maximum depressive symptoms (Ge et al. 2001; Natsuaki et al. 2009), but contradicted other research suggesting that females have an earlier age of maximum depressive symptoms compared to males (Edwards et al. 2014). However, the authors here did not use the same number of measurements, used a quadratic polynomial model and only had data that went up to approximately 18 years old. Future research should examine depressive symptoms regularly around these ages to see if the critical periods identified are similar across contexts. Such research will inform services, schools and parents about the nature of depression and how to prevent and treat it around these ages.

Still, it is not clear why females on average have higher trajectories of depressive symptoms and why females and males differ in their ages of peak velocity of depressive symptoms. One explanation is that women tend to experience puberty earlier than men and evidence has suggested that early pubertal timing may be a mechanism responsible for depression and higher depressive symptoms (Joinson et al. 2012; Thapar et al. 2012). Research has also shown that an earlier age of menarche is positively associated with higher depressive symptoms (Joinson et al. 2013; Joinson et al. 2011) and a causal mechanism for greater depressive symptoms (Sequeira et al. 2017). Transitioning through puberty is associated with other psychological and social changes, and individuals who transition early may not have developed the cognitive and emotional skills to combat these changes, and therefore experience lasting effects of depressive symptoms. Likewise, early pubertal changes could result in increased responsiveness to stressors in females, resulting in higher depressive symptoms (Thapar et al. 2012). These findings suggest that individuals with higher starting points, had higher trajectories. Therefore an earlier age of higher depressive symptoms may set an individual up for a higher trajectory which takes longer to recover from. This could explain why females have higher trajectories compared to males, although more research using the timing and changes in pubertal status for both males and females would be needed to substantiate this claim.

Additionally, Angold et al. (1998) found that depression was higher for females between the ages of 9 and 16, and this seemed to coincide with both pubertal status and timing of puberty. Of note, the number of girls with depression in their study was highest at around age 14, which coincides with the age of peak velocity of depressive symptoms in the present study. This suggests there may be some common mechanism at this age that is underpinning depressive symptoms, and how this manifests. The age of peak velocity from the current study tends to match the ages at which both males and females experience puberty and so one possible reason why the current study observed varying ages of peak velocity of depressive symptoms between males and females may be through the role of puberty.

Despite a number of strengths, this study has several limitations that should be highlighted. One limitation that arises with the data used here, and more generally with longitudinal data, is attrition and the role this plays in biasing results towards individuals who respond. The sample size in this study decreased from 7,335 at the first wave of data collection to 3,840 by the eighth occasion opening the possibility to potential attrition bias. Analysis on individuals with depressive symptoms data at both the first and last occasion, compared to those with depressive symptoms data at the first occasion but not the last, revealed differences in the overall symptoms scores and underlying demographics. However, this is consistent with previous research (Edwards et al. 2014), and suggests that studies with attrition could be underestimating the effect of sex differences in trajectories of depressive symptoms as females were more likely to have not responded at the last occasion. Previous studies have also imputed missing data utilising a missing at random approach (MAR) but found that bias due to systematic missingness in ALSPAC is not substantial (Bould et al. 2014; Pearson et al. 2017). The multilevel growth-curves models used in the present study also use FIML to account for missing data and sensitivity analyses revealed that the main effects of this study (i.e., the trajectories and the critical points) were not substantively affected by the inclusion of covariates associated with missing data and attrition bias. Nevertheless, future studies should highlight missing data patterns and attempt to account for data that could potentially be missing not at random.

Another limitation in this study stems from the choice of model used and the assumptions made with multilevel growth-curve models. Modelling trajectories of depressive symptoms appropriately is challenging. A cubic polynomial model was chosen given it is a more parsimonious approach in comparison to splines and fractional polynomials and that it has been used in previous studies that show nonlinearity (Ferro et al. 2015a; Rawana and Morgan 2014). However, alternative approaches such as a restricted cubic spline model may fit the data better at the expense of parsimony. Similarly, a quartic polynomial model may be a better model if the depressive symptoms data continues to rise. Cubic terms (and other polynomials) may also perform poorly at the start and end of the trajectories, as well as potentially producing artificial turns in the data that do not exist (Tilling et al. 2014). Checks were made to ensure that no artificial turns occurred in the data by comparing against other models and the underlying data and descriptive statistics for plausibility. Additionally, the age of peak velocity of depressive symptoms and age of maximum depressive symptoms were calculated well within the range of the trajectories so any bias from potentially mismodelling the start and end of the trajectories is minimised.

A similar limitation in this study is in regard to highlighting variability with the multilevel growth-curve model. A problem with population growth curves like the ones used in the present study is that it is harder to convey how much variability exists across the population, compared to other methods such as latent class growth analysis or growth mixture modelling, which typically stratify population trajectories into multiple subpopulation trajectories. Future research could derive critical periods from latent class analysis to examine if certain groups of trajectories have earlier ages of peak velocity or later times of maximum depressive symptoms. Such research could further highlight variability in critical points across multiple trajectories.

## Conclusion

The nature of trajectories of depressive symptoms between adolescence and young adulthood is not yet clear, but research has suggested that increased levels of depressive symptoms throughout this period are associated with a greater risk of psychopathology and adverse social outcomes in later life (Fergusson et al. 2007; Yaroslavsky et al. 2013). It is important to examine the nature of depressive symptoms and identify critical periods that could potentially be used to target a stage of development where depressive symptoms are increasing at the fastest rate. A greater understanding of these two facets would aid in developing and improving treatments and interventions. This study examined trajectories of depressive symptoms between males and females in a large UK based population cohort over multiple stages of development. Using multilevel growth-curve models, the findings suggested that females on average were associated with steeper trajectories of depressive symptoms compared to males, indicating they were more likely to experience higher depressive symptoms for longer. Importantly, the age at which depressive symptoms were increasing most rapidly were much earlier for females on average compared to males. Whilst the mechanisms underpinning this sex difference are not entirely understood, pubertal status and the timing of pubertal status could play a role in explaining why females on average have higher trajectories and commence on these trajectories earlier than males. Calculating the age of peak velocity of depressive symptoms is a potentially useful tool for exploring how depressive symptoms are changing and at what age they are increasing most rapidly, which may have consequences downstream. If this can be used for clinical purposes, it may be possible to treat individuals at this age, which may help reduce depressive symptoms or depression at a later stage.

## Supplementary Information

Supplementary Information

## Footnotes

1 Age was centered to approximately 16 years to make the intercept more interpretable and is common practise in multilevel growth-curve modelling studies (Rawana & Morgan, 2014; Singer & Willett, 2003). To achieve this, the mean age of all the age measurements was calculated and subtracted this from approximately 16 (15.82).
