# Supplementary material for: Identifying Critical Points of Trajectories of Depressive Symptoms from Childhood to Young Adulthood
Source: J Youth Adolesc. 2019 Jan 22;48(4):815–27. doi: 10.1007/s10964-018-0976-5 (PMC6441403; doi:10.1007/s10964-018-0976-5)
Supplement: Supplementary file 1 — Supplementary Information [file 10964_2018_976_MOESM1_ESM.docx]

**Identifying Critical Points of Trajectories of Depressive Symptoms from Childhood to Young Adulthood - Supplementary Materials**

**Model equation**

Reconsider equation 1 in the manuscript:

$$y_{ij}=\beta_{0}+\beta_{1}t_{ij}+\beta_{2}t_{ij}^{2}+\beta_{3}t_{ij}^{3}+\beta_{4}x_{1j}+\beta_{5}x_{1j}t_{ij}+\beta_{6}x_{1j}t_{ij}^{2}+\beta_{7}x_{1j}t_{ij}^{3}$$

$$+u_{0j}+u_{1j}t_{ij}+u_{2j}t_{ij}^{2}+u_{3j}t_{ij}^{3}+e_{ij}$$

(Supplementary equation 1)

where $y_{ij}$ is the depressive symptom score and $t_{ij}$ is the age (centred around 16 years, the approximate sample mean) for individual $j$ at occasion $i$, $x_{1j}$ is a dummy variable for being female, and $u_{0j}$, $u_{1j}$, $u_{2j}$, and $u_{3j}$ are the random linear, quadratic and cubic effects, respectively, and $e_{ij}$ is the occasion-specific residual.

**Predicted depressive symptom scores**

The predicted depressive symptom score for individual $j$ at occasion $i$ is given by

$$\hat{y}_{ij}=\beta_{0}+\beta_{1}t_{ij}+\beta_{2}t_{ij}^{2}+\beta_{3}t_{ij}^{3}+\beta_{4}x_{1j}+\beta_{5}x_{1j}t_{ij}+\beta_{6}x_{1j}t_{ij}^{2}+\beta_{7}x_{1j}t_{ij}^{3}$$

$$+u_{0j}+u_{1j}t_{ij}+u_{2j}t_{ij}^{2}+u_{3j}t_{ij}^{3}$$

(Supplementary equation 2)

The mean male and female predicted depressive symptom score at a given age $t_{ij}$ can be obtained by setting all random effects to 0 and by setting $x_{1j}$ to 0 or 1 accordingly.

**Velocity**

Differentiating supplementary equation 1 with respect to $t_{ij}$ gives the velocity (rate of change) in depressive symptoms

$$\frac{\partial y_{ij}}{\partial t_{ij}}=\beta_{1}+2\beta_{2}t_{ij}+3\beta_{3}t_{ij}^{2}+\beta_{5}x_{1j}+2\beta_{6}x_{1j}t_{ij}+3\beta_{7}x_{1j}t_{ij}^{2}+u_{1j}+2u_{2j}t_{ij}+3u_{3j}t_{ij}^{2}$$

(Supplementary equation 3)

The mean male and female velocities at a given age $t_{ij}$ can be obtained by setting all random effects to 0 and by setting $x_{1j}$ to 0 or 1 accordingly.

**Acceleration**

Differentiating supplementary equation 1 twice with respect to $t_{ij}$ gives the acceleration in depressive symptoms

$$\frac{\partial^{2}y_{ij}}{\partial t_{ij}^{2}}=2\beta_{2}+6\beta_{3}t_{ij}+2\beta_{6}x_{ij}+6\beta_{7}x_{ij}t_{ij}+2u_{2j}+6u_{3j}t_{ij}$$

(Supplementary equation 4)

The mean male and female accelerations at a given age $t_{ij}$ can be obtained by setting all random effects to 0 and by setting $x_{1j}$ to 0 or 1 accordingly.

**Age of peak velocity**

Setting supplementary equation 4 to zero and rearranging gives the age of peak velocity of depressive symptoms:

$$t_{ij,\mathrm{APV}}=-\frac{2\beta_{2}+2\beta_{6}x_{1j}+2u_{2j}}{6\beta_{3}+6\beta_{7}x_{1j}+6u_{3j}}$$

(Supplementary equation 5)

The predicted depressive symptom scores evaluated at $t_{ij,\mathrm{APV}}$ can be found by substituting $t_{ij,\mathrm{APV}}$ into supplementary equation 2.

The mean male and female ages of peak velocity can be obtained by setting all random effects to 0 and by setting $x_{1j}$ to 0 or 1 accordingly.

**Age of maximum depressive symptoms**

Setting supplementary equation 3 equal to zero and rearranging using the quadratic formulae gives the age of the two turning points (i.e., age at minimum and age at maximum depressive symptoms.

$$t_{ij,\mathrm{AMDS}}=\frac{-2(\beta_{2}+\beta_{6}x_{1j}+u_{2j})\pm\sqrt{{4\left( \beta_{2}+\beta_{6}x_{1j}+u_{2j} \right)}^{2}-12\left( \beta_{1}+\beta_{5}x_{1j}+u_{1j} \right)\left( \beta_{3}+\beta_{7}x_{1j}+u_{3j} \right)}}{6\left( \beta_{3}+\beta_{7}x_{1j}+u_{3j} \right)}$$

(Supplementary equation 6)

The predicted depressive symptom scores evaluated at the two values of $t_{ij,\mathrm{AMDS}}$ can be found by substituting the two values into supplementary equation 2.

The mean male and female ages of maximum depressive symptoms can be obtained by setting all random effects to 0 and by setting $x_{1j}$ to 0 or 1 accordingly.

**Covariates**

Covariates were included based upon previous evidence from the depressive symptoms literature that highlight correlations between depressive symptoms and missing data (Kingsbury et al., 2016; Mahedy et al., 2017; Pearson et al., 2017). These covariates were completed by the participant’s main carer and assessed during the antenatal period. These included: maternal education (coded as ‘A-level or higher’, ‘O-level’ or ‘<O-level’), maternal social class (coded as ‘Professional occupations or managerial and technical occupations’ or ‘Skilled non-manual occupations, skilled manual occupations, partly-skilled occupations and unskilled occupations’, parity (whether the study child was 1^st^/2^nd^/3^rd^ born or greater), housing tenure (coded as ‘Mortgaged or owned’, ‘Privately rented’ or ‘Subsided rented’), financial difficulties (yes/no), maternal smoking in pregnancy (yes/no), maternal prenatal depression (yes/no) and maternal postnatal depression (yes/no). A binary indicator of the SMFQ source was also included as a covariate (clinic/questionnaire).

Our results were robust to the inclusion of these covariates that are associated with missing data (see Supplementary tables 3-7 and Supplementary figure 4). The total sample that included all the covariates was 6,097 individuals, resulting in 27,952 measurements (12,362 male/15,590 female).

| Supplementary Table 1. Correlational matrix between Short Mood and Feelings Questionnaire (SMFQ) assessments. | | | | | | | | |
| --- | --- | --- | --- | --- | --- | --- | --- | --- |
|  | SMFQ1 | SMFQ2 | SMFQ3 | SMFQ4 | SMFQ5 | SMFQ6 | SMFQ7 | SMFQ8 |
| SMFQ1 | 1 | . | . | . | . | . | . | . |
| SMFQ2 | 0.449 | 1 | . | . | . | . | . | . |
| SMFQ3 | 0.406 | 0.624 | 1 | . | . | . | . | . |
| SMFQ4 | 0.075 | 0.184 | 0.248 | 1 | . | . | . | . |
| SMFQ5 | 0.143 | 0.244 | 0.311 | 0.347 | 1 | . | . | . |
| SMFQ6 | 0.081 | 0.158 | 0.192 | 0.456 | 0.34 | 1 | . | . |
| SMFQ7 | 0.068 | 0.143 | 0.199 | 0.406 | 0.323 | 0.472 | 1 | . |
| SMFQ8 | 0.037 | 0.116 | 0.169 | 0.372 | 0.313 | 0.439 | 0.546 | 1 |

| Supplementary Table 2. Participant demographics for individuals with SMFQ at both 10 and 22 years compared to only SMFQ at 10 years. | | | | |
| --- | --- | --- | --- | --- |
|  | Has SMFQ at 10 and 22 Years | Has SMFQ at 10 but not 22 years | Test Statistic | *p* |
| **SMFQ** |  |  |  |  |
| Mean SMFQ (SD) *n* = 7,335 | 3.78 (3.33) | 4.23 (3.62) | *t*(7333) = 5.38 | *p* < .001 |
| Below Threshold at SMFQ T1 *n* (%) | 2,937 (95.5) | 3,962 (93.1) | $x$^2^ = 18.43 | *p* < .001 |
| Above Threshold at SMFQ T1 *n* (%) | 140 (4.5) | 296 (6.9) |  |  |
| **Sex** |  |  |  |  |
| Males *n* (%) | 1,087 (35.3) | 2,518 (59.1) | $x$^2^ = 405.14 | *p* < .001 |
| Females *n* (%) | 1,990 (64.7) | 1,740 (40.9) |  |  |
| **Maternal Education** |  |  |  |  |
| A Level or Higher *n* (%) | 1,466 (50.8) | 1,382 (36.2) | $x$^2^ = 196.98 | *p* < .001 |
| O Level *n* (%) | 986 (34.2) | 1,392 (36.5) |  |  |
| < O Level *n* (%) | 434 (15%) | 1,042 (27.3) |  |  |
| **Maternal Socioeconomic Status** |  |  |  |  |
| Professional/Managerial/Technical *n* (%) | 1,201 (46.8) | 1,218 (37.7) | $x$^2^ = 48.74 | *p* < .001 |
| Skilled non-manual or lower *n* (%) | 1,363 (53.2) | 2,010 (62.3) |  |  |
| **Parity** |  |  |  |  |
| First Born *n* (%) | 1,413 (49.4) | 1,686 (43.6) | $x$^2^ = 24.77 | *p* < .001 |
| Second Born *n* (%) | 1,000 (34.9) | 1,442 (37.3) |  |  |
| Third Born + *n* (%) | 450 (15.7) | 737 (19.1) |  |  |
| **Housing** |  |  |  |  |
| Mortgaged/Owned *n* (%) | 2,505 (89.7) | 3,101 (81.9) | $x$^2^ = 84.51 | *p* < .001 |
| Private Rented *n* (%) | 123 (4.4) | 227 (6) |  |  |
| Subsidised Rented *n* (%) | 164 (5.9) | 457 (12.1) |  |  |
| **Financial Difficulties** |  |  |  |  |
| No Financial Difficulties *n* (%) | 2,334 (82.7) | 2,765 (74.5) | $x$^2^ = 62.27 | *p* < .001 |
| Yes Financial Difficulties *n* (%) | 490 (17.3) | 947 (25.5) |  |  |
| **Smoking During Pregnancy** |  |  |  |  |
| No Prenatal Smoking *n* (%) | 2,514 (86.4) | 3,113 (79.4) | $x$^2^ = 55.75 | *p* < .001 |
| Yes Postnatal Smoking *n* (%) | 396 (13.6) | 806 (20.6) |  |  |
| **Depression Pre-Pregnancy** |  |  |  |  |
| No Prenatal Depression *n* (%) | 2,375 (87.5) | 3,051 (83.8) | $x$^2^ = 405.14 | *p* < .001 |
| Yes Prenatal Depression *n* (%) | 339 (12.5) | 591 (16.2) |  |  |
| **Depression Pre-Pregnancy** |  |  |  |  |
| No Postnatal Depression *n* (%) | 2,515 (89.6) | 3,246 (87.3) | $x$^2^ = 405.14 | *p* = .004 |
| Yes Postnatal Depression *n* (%) | 291 (10.4) | 472 (12.7) |  |  |

|  |  | | | | | | | | |
| --- | --- | --- | --- | --- | --- | --- | --- | --- | --- |
|  | Quadratic | | | Cubic | | | Quartic | | |
| Parameter | Estimate | Std. Error | *p*-value | Estimate | Std. Error | *p*-value | Estimate | Std. Error | *p*-value |
| $\beta_{0}$ - Intercept | 4.538 | 0.069 | <.001 | 4.568 | 0.069 | <.001 | 4.736 | 0.082 | <.001 |
| $\beta_{1}$- Age (slope) | 0.135 | 0.01 | <.001 | 0.357 | 0.022 | <.001 | 0.361 | 0.022 | <.001 |
| $\beta_{2}$- Age^2 (acceleration) | 0.002 | 0.002 | 0.308 | 0.012 | 0.002 | <.001 | -0.026 | 0.009 | 0.003 |
| $\beta_{3}$- Age^3 (cubic change) | - | - | - | -0.007 | 0.001 | <.001 | -0.009 | 0.001 | <.001 |
| $\beta_{4}$- Female | 2.222 | 0.095 | <.001 | 2.132 | 0.095 | <.001 | 2.328 | 0.112 | <.001 |
| $\beta_{5}$- FemalexAge | 0.191 | 0.013 | <.001 | 0.135 | 0.03 | <.001 | 0.169 | 0.031 | <.001 |
| $\beta_{6}$- FemalexAge^2 | -0.053 | 0.003 | <.001 | -0.051 | 0.003 | <.001 | -0.099 | 0.012 | <.001 |
| $\beta_{7}$- FemalexAge^3^ | - | - | - | 0.002 | 0.001 | 0.036 | 0.001 | 0.0002 | <.001 |
| $\beta_{8}$- Age^4^ (quartic change) | - | - | - | - | - | - | 0.001 | 0.0003 | <.001 |
| $\beta_{9}$- FemalexAge^4^ | - | - | - | - | - | - | -0.001 | 0.001 | 0.335 |
| Intercept variance | 12.2 | 0.294 | - | 11.9335 | 0.2918 | - | 14.431 | 0.3885 | - |
| Intercept/Slope covariance | 0.773 | 0.03 | - | 1.2169 | 0.0623 | - | 1.3367 | 0.0736 | - |
| Slope variance | 0.115 | 0.005 | - | 0.4675 | 0.0275 | - | 0.5171 | 0.0281 | - |
| Intercept/Quadratic covariance | -0.149 | 0.007 | - | -0.1172 | 0.0076 | - | -0.4715 | 0.033 | - |
| Slope/Quadratic covariance | -0.004 | 0.001 | - | -0.0055 | 0.002 | - | -0.0243 | 0.0073 | - |
| Quadratic variance | 0.003 | 0.0003 | - | 0.0023 | 0.0003 | - | 0.0363 | 0.0038 | - |
| Intercept/Cubic covariance | - | - | - | -0.0163 | 0.0017 | - | -0.0282 | 0.0023 | - |
| Slope/Cubic covariance | - | - | - | -0.008 | 0.0007 | - | -0.0101 | 0.0008 | - |
| Quadratic/Cubic covariance | - | - | - | 0.0003 | 0.0001 | - | 0.0013 | 0.0003 | - |
| Cubic variance | - | - | - | 0.0001 | 0.00002 | - | 0.0002 | 0.00003 | - |
| Intercept/Quartic covariance | - | - | - | - | - | - | 0.0079 | 0.0007 | - |
| Slope/Quartic covariance | - | - | - | - | - | - | 0.0005 | 0.0002 | - |
| Quadratic/Quartic covariance | - | - | - | - | - | - | -0.0006 | 0.0001 | - |
| Cubic/Quartic covariance | - | - | - | - | - | - | -0.00002 | 0.00001 | - |
| Quartic variance | - | - | - | - | - | - | 0.00001 | 0.000002 | - |
| Female residual variance | 14.481 | 0.173 | - | 13.69 | 0.174 | - | 13.038 | 0.175 | - |
| Male residual variance | 9.765 | 0.141 | - | 9.332 | 0.149 | - | 8.899 | 0.151 | - |
| Female/Male Variance *P* Wald-Test | <.001 | | | <.001 | | | <.001 | | |
| ICC | 0.55 | | | 0.56 | | | 0.62 | | |
| Deviance | 228583.53 | | | 227890.58 | | | 227532 | | |
| AIC | 228611.5 | | | 227930.6 | | | 227586.5 | | |
| BIC | 228731.9 | | | 228102.5 | | | 227818.6 | | |

Supplementary Table 3. Model comparisons between the quadratic, cubic and quartic models.


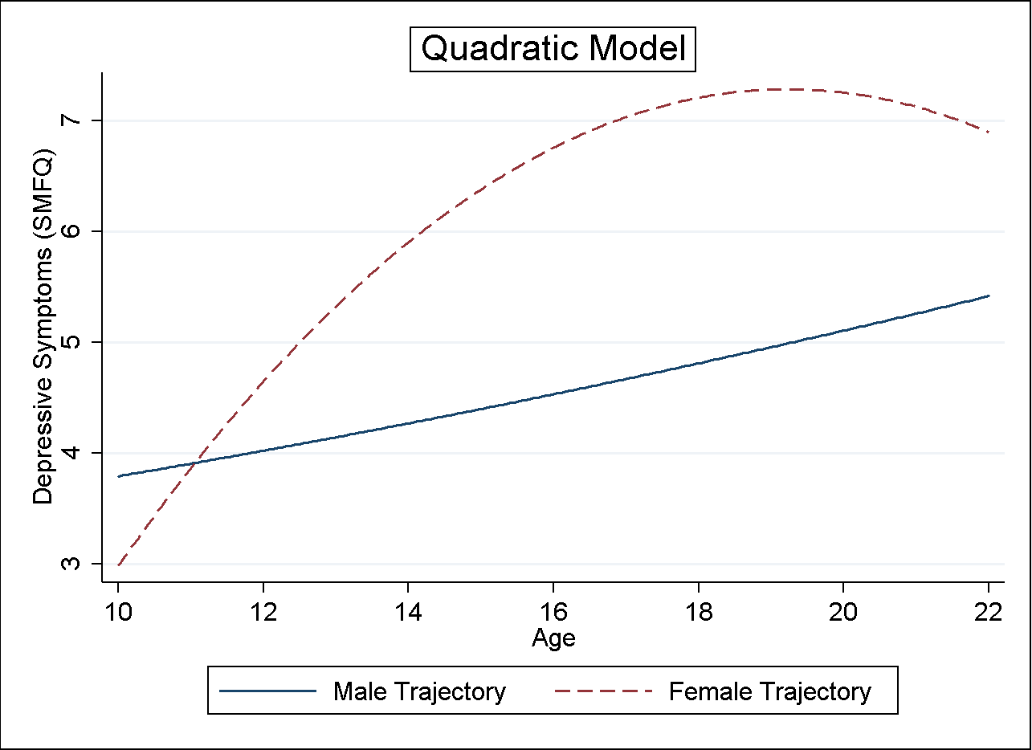
Supplementary figure 1. The quadratic model for males and females.


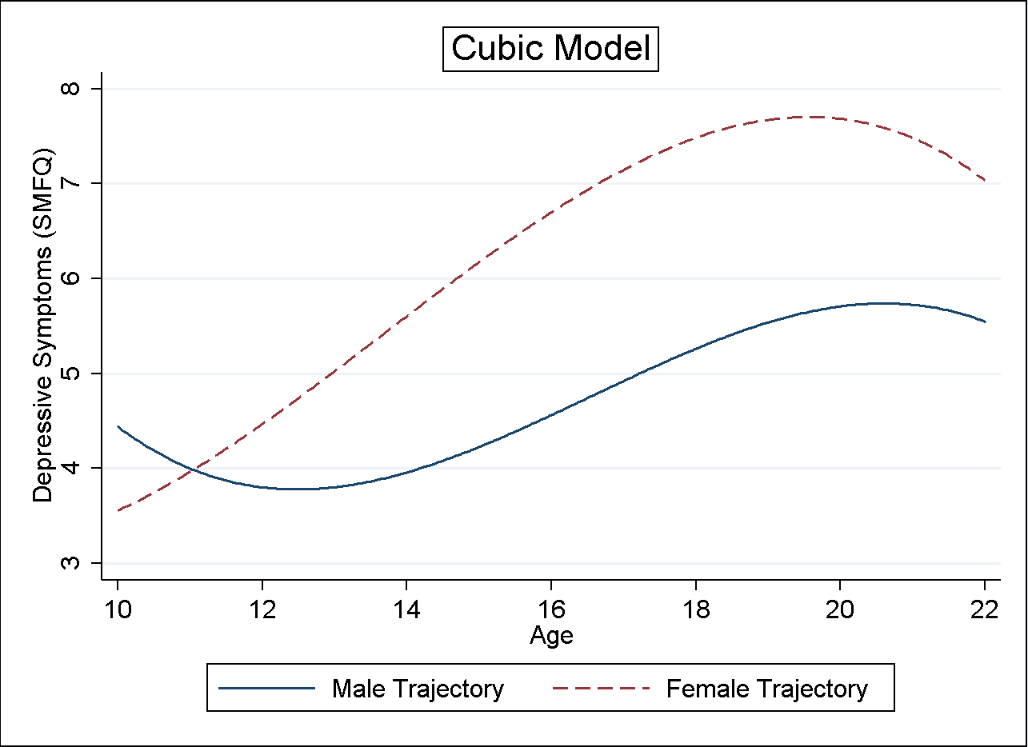
Supplementary figure 2. The cubic model for males and females


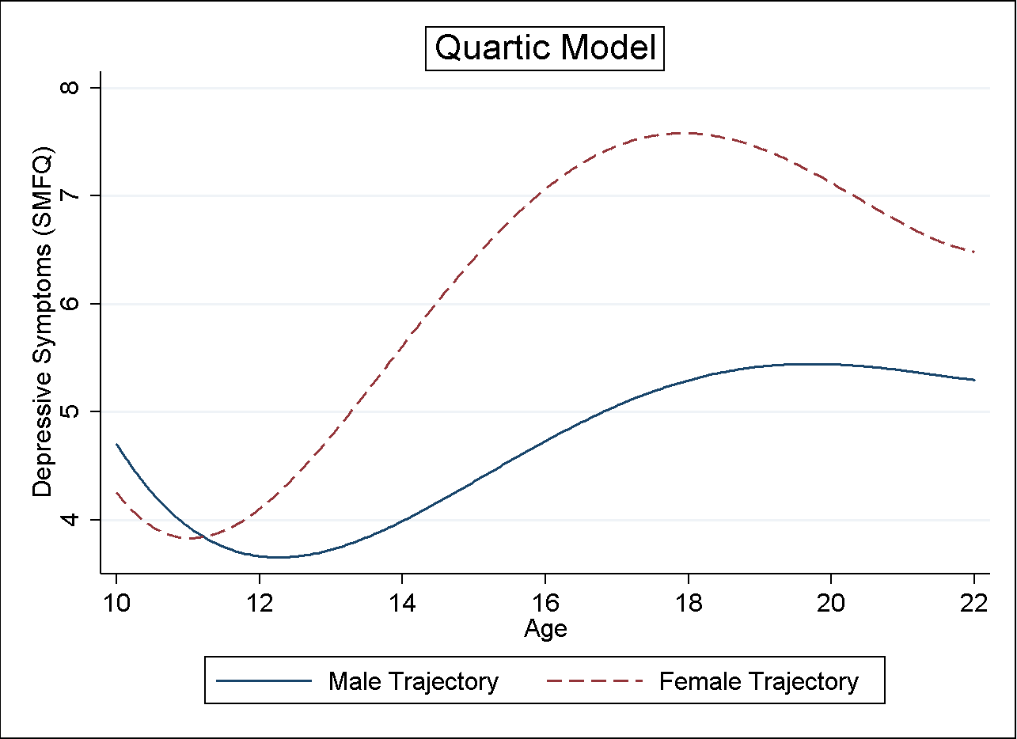
Supplementary figure 3. The quartic model for males and females.

|  | Unadjusted Model (n=9,301) | | | | Adjusted Model (n=6,097) | | |
| --- | --- | --- | --- | --- | --- | --- | --- |
| Parameter | Estimate | | Std. Error | *p*-value | Estimate | Std. Error | *p*-value |
| $\beta_{0}$ - Intercept | 4.568 | 0.069 | | <.001 | 3.997 | 0.113 | <.001 |
| $\beta_{1}$- Age (slope) | 0.357 | 0.022 | | <.001 | 0.380 | 0.027 | <.001 |
| $\beta_{2}$- Age^2 (acceleration) | 0.012 | 0.002 | | <.001 | 0.013 | 0.003 | <.001 |
| $\beta_{3}$- Age^3 (cubic change) | -0.007 | 0.001 | | <.001 | -0.007 | 0.001 | <.001 |
| $\beta_{4}$- Female | 2.132 | 0.095 | | <.001 | 1.99 | 0.112 | <.001 |
| $\beta_{5}$- FemalexAge | 0.135 | 0.03 | | <.001 | 0.129 | 0.036 | <.001 |
| $\beta_{6}$- FemalexAge^2 | -0.051 | 0.003 | | <.001 | -0.051 | 0.004 | <.001 |
| $\beta_{7}$- FemalexAge^3 | 0.002 | 0.001 | | 0.036 | 0.001 | 0.001 | 0.148 |
| Measurement | - | - | | - | 0.252 | 0.073 | 0.001 |
| Mat Edu - O Level | - | - | | - | -0.019 | 0.097 | 0.842 |
| Mat Edu - <O Level | - | - | | - | -0.161 | 0.121 | 0.181 |
| Mat Sclass - III-V | - | - | | - | -0.053 | 0.091 | 0.563 |
| 2nd Born | - | - | | - | 0.012 | 0.085 | 0.887 |
| 3rd Born+ | - | - | | - | 0.355 | 0.114 | 0.002 |
| Housing - Private Rented | - | - | | - | 0.317 | 0.184 | 0.085 |
| Housing - Subsidised Rented | - | - | | - | 0.291 | 0.164 | 0.076 |
| Financial Difficulties | - | - | | - | 0.595 | 0.101 | <.001 |
| Smoked During Pregnancy | - | - | | - | 0.829 | 0.11 | <.001 |
| Depression Pre-Pregnancy | - | - | | - | 0.641 | 0.119 | <.001 |
| Depression Post-Pregnancy | - | - | | - | 0.403 | 0.131 | 0.002 |
| Intercept variance | 11.9335 | 0.2918 | | - | 11.1721 | 0.3315 | - |
| Intercept/Slope covariance | 1.2169 | 0.0623 | | - | 1.0772 | 0.0707 | - |
| Slope variance | 0.4675 | 0.0275 | | - | 0.4458 | 0.0314 | - |
| Intercept/Quadratic covariance | -0.1172 | 0.0076 | | - | -0.1197 | 0.0088 | - |
| Slope/Quadratic covariance | -0.0055 | 0.0020 | | - | -0.0044 | 0.0024 | - |
| Quadratic variance | 0.0023 | 0.0003 | | - | 0.0026 | 0.0004 | - |
| Intercept/Cubic covariance | -0.0163 | 0.0017 | | - | -0.0129 | 0.0019 | - |
| Slope/Cubic covariance | -0.008 | 0.0007 | | - | -0.0075 | 0.0008 | - |
| Quadratic/Cubic covariance | 0.0003 | 0.0001 | | - | 0.0002 | 0.0001 | - |
| Cubic variance | 0.0001 | 0.00002 | | - | 0.0001 | 0.00002 | - |
| Female residual variance | 13.69 | 0.174 | | - | 13.175 | 0.2 | - |
| Male residual variance | 9.332 | 0.149 | | - | 9.372 | 0.175 | - |
| Female/Male Variance *P* Wald-Test | <.001 | | | | <.001 | | |
| ICC | 0.56 | | | | 0.54 | | |
| Deviance | 227890.58 | | | | 158562.55 | | |
| AIC | 227930.6 | | | | 158626.5 | | |
| BIC | 228102.5 | | | | 158890.2 | | |

Supplementary Table 4. Full regression coefficients and variances for the unadjusted and adjusted cubic models.

|  | Unadjusted Model (n=5,409) | | | Adjusted Model (n=3,867) | | |
| --- | --- | --- | --- | --- | --- | --- |
| Parameter | Estimate | Std. Error | *p*-value | Estimate | Std. Error | *p*-value |
| $\beta_{0}$ - Intercept | 4.471 | 0.082 | <.001 | 3.955 | 0.126 | <.001 |
| $\beta_{1}$- Age (slope) | 0.356 | 0.024 | <.001 | 0.379 | 0.029 | <.001 |
| $\beta_{2}$- Age^2 (acceleration) | 0.012 | 0.002 | <.001 | 0.013 | 0.003 | <.001 |
| $\beta_{3}$- Age^3 (cubic change) | -0.007 | 0.001 | <.001 | -0.007 | 0.001 | <.001 |
| $\beta_{4}$- Female | 2.090 | 0.110 | <.001 | 1.977 | 0.126 | <.001 |
| $\beta_{5}$- FemalexAge | 0.10 | 0.032 | 0.002 | 0.105 | 0.038 | 0.005 |
| $\beta_{6}$- FemalexAge^2 | -0.05 | 0.003 | <.001 | -0.05 | 0.004 | <.001 |
| $\beta_{7}$- FemalexAge^3 | 0.002 | 0.001 | 0.006 | 0.002 | 0.001 | 0.038 |
| Measurement | - | - | - | 0.25 | 0.075 | 0.001 |
| Mat Edu - O Level | - | - | - | -0.127 | 0.113 | 0.26 |
| Mat Edu - <O Level | - | - | - | -0.240 | 0.149 | 0.107 |
| Mat Sclass - III-V | - | - | - | 0.030 | 0.106 | 0.778 |
| 2nd Born | - | - | - | 0.004 | 0.099 | 0.971 |
| 3rd Born+ | - | - | - | 0.379 | 0.139 | 0.006 |
| Housing - Private Rented | - | - | - | 0.570 | 0.2 | 0.011 |
| Housing - Subsidised Rented | - | - | - | 0.43 | 0.218 | 0.049 |
| Financial Difficulties | - | - | - | 0.442 | 0.124 | <.001 |
| Smoked During Pregnancy | - | - | - | 0.758 | 0.137 | <.001 |
| Depression Pre-Pregnancy | - | - | - | 0.745 | 0.147 | <.001 |
| Depression Post-Pregnancy | - | - | - | 0.646 | 0.160 | <.001 |
| Intercept variance | 11.3809 | 0.3123 | - | 10.6761 | 0.3485 | - |
| Intercept/Slope covariance | 1.1070 | 0.0651 | - | 0.9934 | 0.0732 | - |
| Slope variance | 0.4483 | 0.0277 | - | 0.4376 | 0.0316 | - |
| Intercept/Quadratic covariance | -0.1093 | 0.0079 | - | -0.1114 | 0.0091 | - |
| Slope/Quadratic covariance | -0.004 | 0.002 | - | -0.003 | 0.0024 | - |
| Quadratic variance | 0.0020 | 0.0003 | - | 0.0023 | 0.0004 | - |
| Intercept/Cubic covariance | -0.0140 | 0.0018 | - | -0.011 | 0.0020 | - |
| Slope/Cubic covariance | -0.0077 | 0.0007 | - | -0.0075 | 0.0008 | - |
| Quadratic/Cubic covariance | 0.0003 | 0.0001 | - | 0.0002 | 0.0001 | - |
| Cubic variance | 0.0001 | 0.00002 | - | 0.0001 | 0.00002 | - |
| Female residual variance | 13.567 | 0.183 | - | 13.083 | 0.208 | - |
| Male residual variance | 9.41 | 0.163 | - | 9.31 | 0.187 | - |
| Female/Male Variance *P* Wald-Test | <.001 | | | <.001 | | |
| ICC | 0.55 | | | 0.54 | | |
| Deviance | 183104.28 | | | 132101.03 | | |
| AIC | 183144.3 | | | 132165 | | |
| BIC | 183311.8 | | | 132422.9 | | |

Supplementary Table 5. Sensitivity analysis of full regression coefficients and variances for the unadjusted and adjusted cubic models with at least 4 measurements of SMFQ.

|  | Unadjusted (n=9,301) | | | | | Adjusted (n=6,097) | | | |
| --- | --- | --- | --- | --- | --- | --- | --- | --- | --- |
|  | Males | Females | Difference | *p-value* | Males | | Females | Difference | *p-value* |
| Intercept term for SMFQ | 4.57 (0.07) | 6.7 (0.07) | 2.13 (0.09) | < 0.001 | 3.99 (0.11) | | 5.99 (0.11) | 1.99 (0.11) | < 0.001 |
|  | [4.43, 4.7] | [6.57, 6.83] | [1.95, 2.32] |  | [3.775, 4.218] | | [5.77, 6.20] | [1.77, 2.21] |  |
| Linear term for SMFQ | 0.36 (0.02) | 0.49 (0.02) | 0.14 (0.03) | < 0.001 | 0.379 (.027) | | 0.51 (0.03) | 0.13 (0.04) | < 0.001 |
|  | [0.31, 0.4] | [0.45, 0.53] | [0.08, 0.19] |  | [0.326, 0.433] | | [0.46, 0.56] | [0.06, 0.2] |  |
| Quadratic term for SMFQ | 0.01 (0.002) | -0.04 (0.002) | 0.05 (0.003) | < 0.001 | 0.013 (.003) | | -0.04 (0.003) | 0.05 (0.004) | < 0.001 |
|  | [0.008, 0.2] | [-0.05, -0.03] | [0.05, 0.06] |  | [0.008, 0.018] | | [-0.04, -0.032] | [0.04, 0.06] |  |
| Cubic term for SMFQ | -0.007 (0.001) | -0.01 (.001) | 0.002 (0.001) | 0.04 | -0.007 (.001) | | -0.01 (0.001) | 0.001 (0.001) | 0.15 |
|  | [-0.01, -0.006] | [-0.001, -0.004] | [0.0001, 0.003] |  | [-0.01, -0.006] | | [-0.01, -0.004] | [0.0001, 0.003] |  |

Supplementary Table 6. Comparing parameter estimates and trajectories from the unadjusted and adjusted cubic models. The intercept was centered to age 16 for interpretability. The differences between each term were calculated as follows: the intercept term for males ($\beta_{0}$) *minus* the intercept term for females ($\beta_{0}+\beta_{4}$), the linear term for males ($\beta_{1}$) *minus* the linear term for females ($\beta_{1}+\beta_{5}$), the quadratic term for males ($\beta_{2}$) *minus* the quadratic term for females ($\beta_{2}+\beta_{6}$), the cubic term for males ($\beta_{3}$) *minus* the cubic term for females ($\beta_{3}+\beta_{7}$). Standard errors are given in (parenthesis), 95% confidence intervals are given in [parenthesis].

|  | Unadjusted (n=9,301) | | | | Adjusted (n=6,097) | | | |
| --- | --- | --- | --- | --- | --- | --- | --- | --- |
|  | Males | Females | Difference | *p*-value | Males | Females | Difference | *p*-value |
| Age of Peak Velocity in SMFQ | 16.36 (0.1) | 13.51 (0.32) | 2.86 (0.34) | < 0.001 | 16.44 (0.12) | 13.66 (0.36) | 2.78 (0.37) | < 0.001 |
|  | [16.18, 16.55] | [12.88, 14.14] | [2.2, 3.51] |  | [16.21, 16.67] | [12.97, 14.36] | [2.05, 3.51] |  |
| Age of Maximum SMFQ | 20.42 (0.14) | 19.61 (0.5) | 0.80 (0.55) | 0.14 | 20.68 (0.18) | 19.68 (0.58) | .99 (0.65) | 0.13 |
|  | [20.14, 20.69] | [18.63, 20.6] | [-0.27, 1.88] |  | [20.32, 21.03] | [18.54, 20.82] | [-0.28, 2.27] |  |
| SMFQ at Peak Velocity | 4.76 (0.07) | 5.42 (0.06) | 0.66 (0.1) | < 0.001 | 4.24 (0.12) | 4.77 (0.12) | 0.54 (0.12) | < 0.001 |
|  | [4.62, 4.91] | [5.30, 5.55] | [0.47, 0.85] |  | [4.01, 4.46] | [4.54, 5.0] | [0.31, 0.77] |  |
| SMFQ at Maximum Point | 5.75 (0.1) | 7.7 (0.09) | 1.95 (0.14) | < 0.001 | 5.33 (0.13) | 7.06 (0.13) | 1.73 (0.16) | < 0.001 |
|  | [5.55, 5.95] | [7.52, 7.88] | [1.69, 2.22] |  | [5.07, 5.59] | [6.81, 7.30] | [1.41, 2.04] |  |

Supplementary Table 7. Calculated features from the trajectories from the unadjusted and adjusted cubic models. Standard errors are given in (parenthesis), 95% confidence intervals are given in [parenthesis].

|  | Unadjusted (n=5,409) | | | | Adjusted (n=3,867) | | | |
| --- | --- | --- | --- | --- | --- | --- | --- | --- |
|  | Males | Females | Difference | *p*-value | Males | Females | Difference | *p*-value |
| Age of Peak Velocity in SMFQ | 16.358 (.106) | 13.09 (.451) | 3.267 (.464) | < 0.001 | 16.78 (.124) | 13.702 (.473) | 3.077 (.489) | < 0.001 |
|  | [16.15, 16.566] | [12.206, 13.975] | [2.359, 4.176] |  | [16.536, 17.023] | [12.775, 14.63] | [2.119, 4.035] |  |
| Age of Maximum SMFQ | 20.797 (.146) | 20.519 (.684) | 0.277 (.701) | 0.701 | 20.979 (.179) | 20.528 (.756) | .451 (.81) | 0.578 |
|  | [20.51, 21.083] | [19.179, 21.859] | [-1.136, 1.691] |  | [20.629, 21.329] | [19.047, 22.01] | [-1.136, 2.037] |  |
| SMFQ at Peak Velocity | 4.527 (.083) | 4.9 (.066) | 0.369 (.106) | 0.001 | 4.177 (.127) | 4.564 (.127) | 0.386 (.129) | 0.003 |
|  | [4.364, 4.69] | [4.766, 5.026] | [0.161, 0.577] |  | [3.928, 4.427] | [4.314, 4.813] | [0.133, 0.639] |  |
| SMFQ at Maximum Point | 5.644 (.113) | 7.407 (.098) | 1.764 (.149) | < 0.001 | 5.258 (.146) | 6.891 (.135) | 1.633 (.171) | < 0.001 |
|  | [5.423, 5.865] | [7.215, 7.6] | [1.471, 2.056] |  | [4.973, 5.544] | [6.627, 7.155] | [1.298, 1.968] |  |

Supplementary Table 8. Calculated features from the trajectories from the unadjusted and adjusted cubic models post sensitivity analysis (minimum of 4 measurements included). Standard errors are given in (parenthesis), 95% confidence intervals are given in [parenthesis].

Supplementary
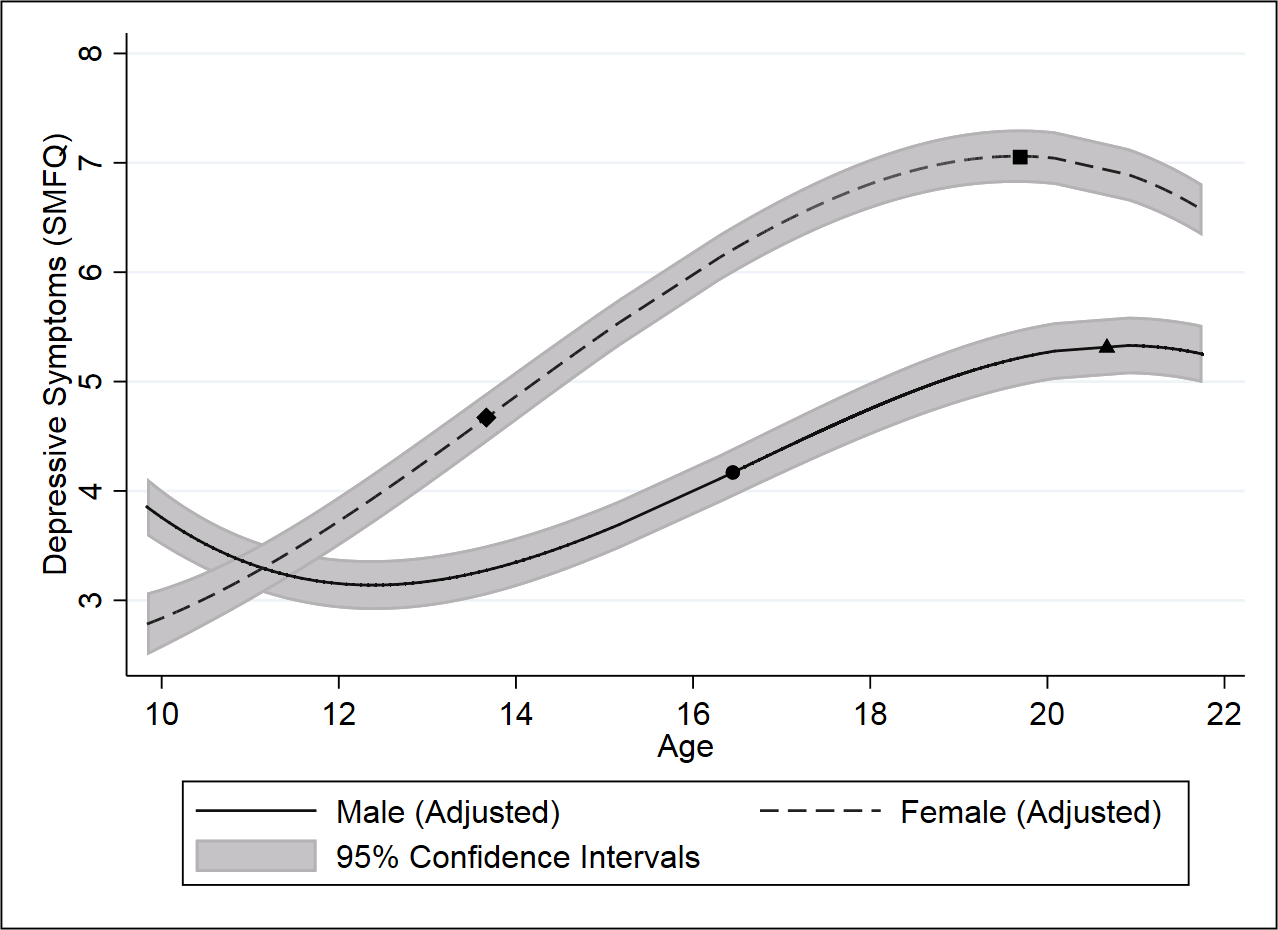
figure 4. Adjusted population trajectories for males and females. SMFQ: Short Mood and Feelings Questionnaire. Features of the trajectories are overlaid with the following terms: ● Male age of peak velocity of depressive symptoms. ▲ Male age of maximum depressive symptoms. ♦ Female age of peak velocity of depressive symptoms. ■ Female age of maximum depressive symptoms.

**Stata Code**

Useful information about multilevel modelling and data preparation can be found here: <https://www.cmm.bris.ac.uk/lemma/>. Lemma is a free to use for academics and contains several tutorials designed to introduce and conduct multilevel modelling in Stata, R, SPSS and MLwiN.

All analyses were conducted using Stata v14 (StataCorp, College Station, TX, USA) using the runmlwin command, which calls MLwiN v2.35 ([www.cmm.bristol.ac.uk/MLwiN/index.shtml](http://www.cmm.bristol.ac.uk/MLwiN/index.shtml)).

For simplicity, we only run the simple unadjusted model to demonstrate how we calculated the features of the trajectories such as age of peak velocity and age of maximum depressive symptoms.

**Once the data has been transformed into long format (you’ll normally have it in wide *format), you can explore the data and ensure you have all the variables needed to run the *actual analysis.*

order ///
subject /// Participant ID
occ /// Occasion number
dep /// Depressive symptoms score
age /// Age at each occasion
female /// The sex indicator with males (0) or females (1)

**You can centre the age variable to make the interpretation easier. From here you can add *your polynomials. We have added 3 terms of age as this matches the analysis conducted in *the manuscript, but you could include fewer/more terms, or explore other functions of age *such as fractional polynomials or splines.*

summarize age
generate age16 = age-15.82151
generate age16_2 = age16^2
generate age16_3 = age16^3

**We have set males to be 0 and females to be 1, so the default age variables will represent *males and the interaction between female and age will represent females.*

generate age16xfem = age16*female
generate age16xfem_2 = age16_2*female
generate age16xfem_3 = age16_3*female

**Set the data to be panel.*

xtset subject occ

**Drop the missing data, i.e., anyone without a dep score. For the sensitivity analysis, we *dropped anyone with less than 3 measurements (not done here).*

sort subject occ
by subject: egen numocc = count(dep)
drop if dep==. | age==.

**Describe and observe the missing data patters.*

xtdes if dep~=.
tab occ

**Running the cubic model in STATA with the runmlwin command that calls MLwiN. MLwiN *requires a constant to be generated and a path specified in STATA. We also want to know *about model fit and correlations between our parameters so we specify additional *commands. We save the estimates for use in calculating the trajectory features later.*

gen cons = 1
global MLwiN_path C:\Program Files /// (x86)\MLwiN\v2.35\i386\MLwiN.exe

sort subject age

runmlwin dep cons age16 age16_2 age16_3 female age16xfem ///
age16_2xfem age16_3xfem, ///
level2(subject: cons age16 age16_2 age16_3) ///
level1(age: male female, diagonal) ///
nopause

runmlwin, corr
estat ic
estimates save “unadjusted.ster”

**You can calculate the male and female variance and see if they differ. You can do this by *telling STATA to return the parameters that are stored in the local memory. Then take these *parameters into your equations.*

ereturn list
matrix list e(b)

nlcom (Male_VPC_xis0: [RP2]var(cons)/([RP2]var(cons) ///
+ [RP1]var(male)))
test [RP1]var(female) = [RP1]var(male)

**You can plot the trajectories here using the predict command.*

predict predcub
sort age
line predcub age if female==1, lpattern(solid) || ///
line predcub2 age if female==0, lpattern(dash)||, ///
ytitle("Depressive Symptoms Score") ///
legend(label(1 "Female (Unadjusted)") ///
label(2 "Male (Unadjusted)"))

**You can compare the male and female trajectories using the delta method. First compare *the intercepts (which are centered to approximately 16 years old).*

estimates use "unadjusted.ster"
nlcom ///
(male: _b[cons]) ///
(female: _b[cons] + _b[female]) ///
, post
nlcom (difference: _b[male] - _b[female])

**You can then compare sex differences for the linear, quadratic and cubic terms.*

estimates use "unadjusted.ster"

nlcom ///
(male: _b[age16]) ///
(female: _b[age16] + _b[age16xfem]) ///
, post
nlcom (difference: _b[male] - _b[female])

estimates use "unadjusted.ster"

nlcom ///
(male: _b[age16_2]) ///
(female: _b[age16_2] + _b[age16_2xfem]) ///
, post
nlcom (difference: _b[male] - _b[female])

estimates use "unadjusted.ster"

nlcom ///
(male: _b[age16_3]) ///
(female: _b[age16_3] + _b[age16_3xfem]) ///
, post
nlcom (difference: _b[male] - _b[female])

**Calculating the age of peak velocity of depressive symptoms can be done by using the *parameters stored by STATA, the mean age and using supplementary equation 5.*

estimates use "unadjusted.ster"

ereturn list
matrix list e(b)

nlcom ///
(male: 15.82151 +(-_b[age16_2]/(3*_b[age16_3]))) ///
(female: 15.82151 + (-(_b[age16_2] + ///
_b[age16_2xfem])/(3*(_b[age16_3] ///
+ _b[age16_3xfem])))) ///
, post
nlcom (difference: _b[male] - _b[female])

**Calculating the age of maximum depressive symptoms can be done by using the parameters *stored by STATA, the mean age and using supplementary equation 6.*

estimates use "unadjusted.ster"

nlcom ///
(males: (-2*_b[age16_2] ///
- (sqrt(4*(_b[age16_2]^2) ///
- (12*(_b[age16_3]*_b[age16]))))) ///
/ (6*_b[age16_3]) + 15.82151) ///
(females: (-2*(_b[age16_2]+_b[age16_2xfem]) ///
- (sqrt((4*((_b[age16_2]^2)+(_b[age16_2xfem]^2))) ///
- ///
(12*(_b[age16_3]*_b[age16])+(_b[age16_3xfem]* ///
_b[age16xfem]))))) ///
/ (6*(_b[age16_3]+_b[age16_3xfem])) + 15.82151) ///
, post
nlcom (difference: _b[male] - _b[female])

**Calculating the depressive symptoms score at the age of peak velocity of depressive *symptoms can be done by using the parameters stored by STATA, the mean age subtracted *from the age of peak velocity of depressive symptoms and using supplementary equation 2.*

estimates use "unadjusted.ster"

scalar tm = 16.36378 - 15.82151
scalar tf = 13.50909 - 15.82151

nlcom ///
(male: _b[cons] + _b[age16]*tm + _b[age16_2]*tm^2 ///
+ _b[age16_3]*tm^3) ///
(female: _b[cons] + _b[age16]*tf + _b[age16_2]*tf^2 ///
+ _b[age16_3]*tf^3 ///
+ _b[female] + _b[age16xfem]*tf + _b[age16_2xfem]*tf^2 ///
+ _b[age16_3xfem]*tf^3) ///
, post
nlcom (difference: _b[male] - _b[female])

**Calculating the depressive symptoms score at age of maximum depressive symptoms can be *done by using the parameters stored by STATA, the mean age subtracted from the age of *maximum depressive symptoms and using supplementary equation 2.*

estimates use "unadjusted.ster"

scalar tm = 20.47192 - 15.82151
scalar tf = 19.89151 - 15.82151

nlcom ///
(male: _b[cons] + _b[age16]*tm + _b[age16_2]*tm^2 ///
+ _b[age16_3]*tm^3) ///
(female: _b[cons] + _b[age16]*tf + _b[age16_2]*tf^2 ///
+ _b[age16_3]*tf^3 ///
+ _b[female] + _b[age16xfem]*tf + _b[age16_2xfem]*tf^2 ///
+ _b[age16_3xfem]*tf^3) ///
, post
nlcom (difference: _b[male] - _b[female])

**Supplementary References**

Kingsbury, M., Weeks, M., MacKinnon, N., Evans, J., Mahedy, L., Dykxhoorn, J., & Colman, I. (2016). Stressful Life Events During Pregnancy and Offspring Depression: Evidence From a Prospective Cohort Study. *J Am Acad Child Adolesc Psychiatry, 55*(8), 709-716 e702. doi:10.1016/j.jaac.2016.05.014

Mahedy, L., Hammerton, G., Teyhan, A., Edwards, A. C., Kendler, K. S., Moore, S. C., . . . Heron, J. (2017). Parental alcohol use and risk of behavioral and emotional problems in offspring. *PLoS One, 12*(6), e0178862. doi:10.1371/journal.pone.0178862

Pearson, R. M., Campbell, A., Howard, L. M., Bornstein, M. H., O'Mahen, H., Mars, B., & Moran, P. (2017). Impact of dysfunctional maternal personality traits on risk of offspring depression, anxiety and self-harm at age 18 years: a population-based longitudinal study. *Psychological Medicine*, 1-11. doi:10.1017/s0033291717001246
